# Liver iron susceptometry is different in patients with transfusion dependent thalassemia and sickle cell disease compared with hemochromatosis

**DOI:** 10.1016/j.bbrep.2025.102340

**Published:** 2025-11-12

**Authors:** Roland Fischer, Marcela G. Weyhmiller, Ellen B. Fung, Zahra Pakbaz, Doug N. Paulson, Elliott P. Vichinsky, Peter Nielsen, Paul R. Harmatz

**Affiliations:** a UCSF Benioff Children's Hospital Oakland, Oakland, CA, USA; b UC Irvine School of Medicine, Irvine, CA, USA; c Tristan Technologies, San Diego, CA, USA; d Department of Diagnostic and Interventional Radiology, University Medical Center Hamburg-Eppendorf, Germany

**Keywords:** Iron, Magnetic susceptibility, Ferritin, Hemosiderin, Thalassemia, Hemochromatosis

## Abstract

Biomagnetic liver susceptometry (BLS) is a non-invasive method to quantify liver iron concentration (LIC) based on a universal iron-specific paramagnetic susceptibility (χ_Fe_). In a blinded prospective study validating in vivo LIC by SQUID biosusceptometry (BLS) with in vitro LIC by biopsies, unexplainable discrepancies were evident between the two methodologies. Thus, a re-analysis of relevant data was performed questioning the universal character of χ_Fe_ in liver tissue.

The magnetic volume susceptibility difference relative to water was measured by BLS, and the LIC was assayed by ICP-MS in 40 dry weight fresh-tissue and paraffin-embedded biopsy samples in patients with transfusion dependent thalassemia (TDT) and sickle cell disease (SCD). The wet-to-dry weight ratio was confirmed as 4.1 ± 0.7. Retrospectively, χ_Fe_ was re-analyzed from historic wet-weight LIC in 37 patients with hereditary hemochromatosis (HFE-1).

The ratio of in vivo volume susceptibility difference Δχ to wet-weight LIC resulted in significantly different iron-specific mass susceptibilities χ_Fe_ in patients with TDT, SCD, and HFE-1. From regression analysis of dry-weight LIC versus Δχ(BLS) in patients with TDT & SCD (r^2^ = 0.89), we could derive a susceptibility of χ_Fe_ = (0.96 ± 0.03)⋅10^−6^m^3^/kg and χ_Fe_ = (1.48 ± 0.03)⋅10^−6^m^3^/kg in patients with HFE-1 (r^2^ = 0.99). The discrepancy in former LIC by BLS can be resolved with a factor of 1.5 still using the traditional wet-to-dry weight ratio of 4. Given these findings, it is recommended that for noninvasive magnetic iron measurements, different magnetic susceptibilities be applied to patients with transfusional siderosis (TDT, SCD) compared with hereditary hemochromatosis, and presumably also for other iron loading disorders.

## Highlights

• Liver iron concentration in thalassemia, sickle-cell disease, hemochromatosis.
• Wet-to-dry weight of 4.1 in liver tissue reconfirmed.
• Liver susceptometry in thalassemia demands a lower magnetic susceptibility.
• Hemosiderin and ferritin iron have different susceptibilities in hemochromatosis.
• Magnetic mass susceptibility of 1.0·10^−6^ m^3^/kg should be used in thalassemia.

## 1 Introduction

Patients with iron overload caused by chronic blood transfusion (thalassemia, sickle cell disease, or other transfusion dependent hemoglobinopathies) or by increased iron absorption (hereditary hemochromatosis), are at risk from iron toxicity. If not adequately treated by iron depleting therapies (chelators, phlebotomy), patients can develop multi-organ failure: including liver cirrhosis, endocrinopathy, or cardiac failure [1]. Serum ferritin is widely used to monitor iron stores since it correlates with liver iron concentration (LIC). Using ferritin alone, can be misleading [2]. Total body iron stores are reflected by LIC [3,4], therefore, LIC is routinely assessed to monitor iron overload in response to iron depletion therapies. Originally, chemical quantification of iron in liver tissue samples collected by biopsies was used to monitor iron overload. Frequency of performing biopsies is limited by cost, risk, and invasiveness (pain, bleeding), therefore noninvasive methods have been developed to allow for safer and more frequent LIC assessment in individual patients. In 1967, Bauman and Harris demonstrated that the difference between the diamagnetic susceptibility of water in the body's tissues and the paramagnetic susceptibility of the hemosiderin/ferritin iron complex in the liver could be used to quantify LIC [5]. Since then, several groups explored the application of biomagnetic liver susceptometry (BLS) technology using low-temperature (liquid helium) SQUID (Superconducting QUantum Interference Device) biosusceptometers for the noninvasive quantification of LIC in humans [[6], [7], [8], [9], [10]]. High operating costs, low environmental magnetic noise requirements, and a certain level of operation expertise has prevented a widespread adoption of SQUID-based technology.

Physico-chemical liver iron measurements are performed in small samples of tissue (about 10–15 mm^3^) from biopsies, which are processed prior to iron quantification. Alternative noninvasive techniques to assess LIC in vivo are increasingly used. Quantitative magnetic resonance imaging (MRI) assesses iron concentration in relatively large liver portions in the hydrated or wet state, while relying on calibration studies in small biopsy or autopsy samples [[11], [12], [13]].

BLS technique depends on physical properties, such that biosusceptometers are calibrated using the magnetic volume susceptibility, χ, of any standard object (phantom) of well-known composition and geometry [8,14]. In vivo LIC is calculated relying on the magnetic susceptibility of the hemosiderin/ferritin iron complex (χ_Fe_). For this mass susceptibility, a value of χ_Fe_ = 1.524 ⋅10^−6^ m^3^/kg [[5], [6], [7], [8], [9],[14], [15], [16]] was independently used representing the underlying iron overload disease (hemochromatosis, thalassemia, etc.). Ferritin iron-specific magnetic mass susceptibilities were measured in three autopsy livers from patients with Diamond-Blackfan anemia in the range of (1.59–1.67)⋅10^−6^m^3^/kg, however, hemosiderin iron susceptibilities were observed in the same livers in a relatively large range of (1.15–2.09)⋅10^−6^m^3^/kg [15]. An overview of iron-specific magnetic susceptibility measurements between 1943 and 2012 was compiled [17].

LIC from BLS is reported as microgram (milligram or micromol) iron per gram liver wet weight, and is not comparable to dry weight liver biopsy without a conversion factor. Initial studies of BLS suggested a conversion factor (f_wdr_) being equal to the wet-to-dry weight ratio (WDR) of 3.46, which assumed a water content of 71.1 % for the liver [7]. However, studies in iron overloaded patients have shown variability in the WDR obtained from biopsies suggesting that WDR may depend on the processing technique and the size of the sample obtained [18]. Currently, a WDR of 4.1 is a well-accepted reference value [19,20].

A blinded, prospective study of in vivo LIC from a SQUID biosusceptometer was compared with in vitro LIC from fresh-tissue (FT) biopsies and from widely used paraffin-embedded biopsies (PB). From the wet and dry weight of biopsy samples, conversion factors were calculated to compare LIC by BLS with LIC from liver biopsy. Preliminary data in 2006 reported a conversion factor of 6 in contradiction to a WDR of 3.3 [21]. Further analysis with the complete set of patients confirmed this discrepancy [22].

Therefore, the focus of this work is scrutinizing the previously suggested universal character of the iron-specific paramagnetic liver susceptibility χ_Fe_. We were inspired by the finding of Hackett et al. [23] who measured magnetic susceptibilities in spleen tissue and in a pooled liver sample from four NTDT patients. Especially in the liver, they found a value of 1.14 ⋅10^−6^ m^3^/kg for ferritin iron, which was significantly less than the previously accepted universal value of 1.524 ⋅10^−6^ m^3^/kg for liver ferritin iron [15,16]. Instead of exploiting ac-magnetic susceptometry for in vitro assessment in small tissue samples [23], we utilized in vivo assessment of χ_Fe_ by BLS in large regional liver portions in comparison to iron quantitation in small liver biopsies from the same area.

## 2 Materials and methods

### 2.1 Patients

This study was reviewed and approved by the institutional review board at the UCSF Benioff Children's Hospital Oakland (IRB #2003-026) and fulfilled all ethical board guidelines. Written informed consent was obtained from all patients, and/or their guardian prior to participation in the study. Forty patients with sickle cell disease (SCD: n = 20) and transfusion dependent thalassemia (TDT: n = 20) consented and clinically indicated percutaneous liver biopsies were taken for LIC assessment. All participants had received chronic blood transfusion and chelation treatment by deferoxamine. Mean age was 19.6 years (range, 5–40 y). Each participant underwent biomagnetic liver susceptometry (BLS) and liver biopsy procedure. BLS measurements were compared with the results from fresh-tissue and paraffin-embedded biopsy samples analyzed at Mayo Medical Laboratories, Rochester, MN (MAYO).

In the early nineties a study of 37 HFE = 1 or C282Y(+/+) hemochromatosis patients (age: 22–66 y, f/m: 10/27) with 42 liver biopsies was performed at the University Hospital of Hamburg-Eppendorf (UKE) in cooperation with a nearby specialized hospital (AK Barmbek, University of Hamburg).) This study was supported by grant 01VF8603 of the BMFT (Germany) in order to demonstrate the agreement between LIC by BLS and LIC from wet weight biopsies (LIC: 0.03–10 mg/g_wet weight_, r^2^ = 0.96) [9].

### 2.2 Liver biopsy procedures

In TDT and SCD patients, spring-loaded cutting needles (16g Bard® Monopty® Bard Endoscopic Technologies, Billerica, MA) with a 15 mm^3^ groove for tissue uptake were used for the biopsy procedure and 2 cores were obtained in 2 passes aiming at the same local area of the anterior right liver lobe. Systematically, the liver tissue from 1st biopsy pass was used for histology purposes (paraffin embedding) and after deparaffinization for iron quantitation at MAYO, while the liver tissue from 2nd biopsy pass was used as the fresh-tissue sample for iron quantitation. Fresh-tissue liver biopsy samples were weighed (mean wet weight 10.9 ± 2.2 mg) in pre-weighed trace element free vials. At MAYO, the samples were divided into 2 parts and heat-dried (95 °C for at least 17 h) resulting in a total mean dry weight of 2.7 ± 0.7 mg (range, 1.0–4.0 mg). Dry samples were digested and aliquots (20 μl) were injected into an Inductively Coupled Plasma quadrupole Mass Spectrometer (ICP-MS: Elan DRC II®, PerkinElmer, Boston, USA) for iron measurement.

As described in detail by Butensky et al. [24], the Scheuer and Deugnier scoring system was applied to histologic sections to assess inflammation, fibrosis, and total iron scores, respectively. After obtaining sections for histology, paraffin-embedded liver samples were deparaffinized (mean dry weight 1.1 ± 0.4 mg) according to the method described by Bush et al. [25] and processed as above.

Liver biopsies in HFE-1 patients were obtained by clinically indicated laparoscopy with Menghini needles (Hepafix, Braun, Melsungen, Germany) in pre-weighed trace element free vials with mean net weight of 15.2 ± 10.2 mg wet. In total, forty-two tissue samples were processed from 37 patients using a modified “wet ashing” method described by Zuyderhoudt et al. [19]. In short, after adding 300 μl of H2O, the respective sample was homogenized using a Teflon pistil homogenizer (5000 rev/min, 15 up and down strokes, ice water cooling). Total iron (LIC) was directly measured by atomic absorption spectroscopy (AAS) in 20 μL of the homogenate dissolved in 1 mL matrix solution. From the homogenate, 30 μL was mixed with 1 mL H20, 1 mL Drabkin solution (50 ng/L KCN, 140 mg/L KH2P04), and 0.5 mL of 1 N HCl. After centrifugation, 800 μL of the upper layer was removed and 200 μL of ethanol was added to measure heme iron. For separation of ferritin from hemosiderin, 200 μl of the homogenate was heated to 75 °C for 10 min and then centrifuged. Wet weight iron concentration in the supernatant (soluble iron = ferritin-like iron) or in the rehomogenized pellet (insoluble iron, hemosiderin-like + heme iron) was measured by AAS (HGA 700, PerkinElmer Inc, Waltham, USA). A mean recovery relative to LIC of 96 ± 10 % was obtained for iron cell fractions (hemosiderin, ferritin, and heme iron). The hemosiderin:ferritin iron ratio increased from 1:1 at LIC = 500 μg/g_wet wt_ towards 3:1 beyond 5000 μg/g_wet wt_, as reported elsewhere [17].

### 2.3 Liver susceptometry

The technical details and the measurement procedure of the biosusceptometer (Ferritometer®, Model 5700, Tristan Technologies, San Diego, USA) were described elsewhere [26,27]. More recently, liver susceptometry (BLS) was described in detail in relation to MRI [28]. In short, subjects were placed in a supine position on a motor driven bed with low magnetic signature. Bed-side sonography (EUB-500, Hitachi Med. Systems Inc., Tokyo, Japan) was used for finding an ideal liver position at closest skin-liver distance below the superconducting inhomogeneous magnetic DC-field (max. 35 mT) and gradiometer detection coils. Especially, SQUID voltages (V) and distance (z) were acquired during a 10–13 s breath-hold vertical scan inside a water coupling medium. The data were analyzed within an analytical model [14] of magnetic flux integrals, Φ(z) = ∫**B**_f_ **∙ B**_d_ d^3^**r**, with the magnetic field vector **B**_f_ and the detector coil (“lead”) field vector **B**_d_. SQUID output voltages V(z) were fitted by a linear function of flux integrals (see equation (1)), which were pre-calculated for ellipsoidal liver and cylindrical thorax geometries. The system was calibrated (constant C) against an infinite water sphere versus air with the well-known magnetic volume susceptibility difference Δχ_air-water_ = 9.396**·**10^−6^ [SI units].

(1) Equation 1: V(z) = C (Δχ_liver_**·** Φ_liver_ + Δχ_thorax_**·** Φ_thorax_) + V_system_

From the patients’ body mass index (BMI), the magnetic contribution of the anterior thorax tissue (Δχ_thorax_) was calculated in subjects [29]. Fitting the acquired data to the bi-variate linear regression model (equation (1)), one could derive the in vivo magnetic liver volume susceptibility (also called bulk susceptibility), Δχ_liver_, relative to water. Uncertainties of Δχ_thorax_ calculated from BMI, of z from averaged skin-liver distance by sonography scaling with LIC, and reproducibility errors from 5 scans were assessed and a total standard error of Δχ_liver_ was calculated [14,29]. Liver volume susceptibilities (Δχ_liver_ in ppm or ppb) are related to mass susceptibilities, especially the liver hemosiderin-ferritin iron mass susceptibility (χ_Fe_ in m^3^/kg), by the liver tissue density ρ (=1.05·10^3^ kg/m^3^) [30], equation (2) [17].

(2) Equation 2: Δχ_liver_ = LIC**·** χ_Fe_**·** ρ **/** f_wdr_ + Ø(Δχ_tissue_)

(2a) Equation 2a: LIC_dry wt_ = C_wd_**·** LIC_wet wt_ with the wet-to-dry conversion factor C_wd_, = f_wdr_**·**χ_Fe_^§^ / χ_Fe_

To convert LIC from biopsies in μg/g from *wet weight* into *dry weight* the wet-to-dry weight ratio, f_wdr_, has to be taken into account [17,19], and any χ_Fe_^§^ deviating from the true χ_Fe_., (equation 2a). The term Ø(Δχ_tissue_) characterizes the diamagnetic volume susceptibility difference between liver tissue and water. This difference is much smaller than the imprecision of Δχ_thorax_ estimation (see equation (1)) and may be neglected [23].

The liver susceptometer at the University Hospital Hamburg-Eppendorf (UKE; Biomagnetic Technologies Inc., San Diego, USA) was the prototype for the system at Oakland. The following technical specifications could have affected the performance of the two systems, most important ones were: stronger magnetic field (B_max_) of 37 mT (CHO) vs 20 mT (UKE), higher system and environmental magnetic noise at CHO than at UKE, and smaller water coupling membrane at CHO than at UKE [26,27].

### 2.4 Data analysis and statistics

In order to establish blindness during analysis, 31/40 biosusceptometry results were analyzed and sent to a data depository (Drug and Device Development Co., Inc., Redmond, WA, USA) prior to receiving the fresh-tissue biopsy results from MAYO. All biopsy procedures were performed between 0 and 58 days before biosusceptometry. In six patients, biopsies were repeated after 17–36 months. All results from paraffin-embedded biopsies were received after the BLS procedure. At Hamburg, wet liver tissue samples from laparoscopically performed biopsies were processed for total and fractional iron quantitation by AAS in a specialized biochemistry laboratory.

In iron overload diseases, parameters are typically skewed, thus nonparametric statistics was applied to most of the data: median ± median absolute deviation (MAD), interquartile range (IQR), range (min-max), Spearman rank correlation, and Mann-Whitney *U* Test. Parametric statistics was used for the wet-to-dry weight ratio (mean ± SD) and for regression analysis (COD (r^2^), COV, etc). The following software packages were used for analysis: linear regression was performed by Slide Write Plus for Windows (Version 7, Advanced Graphics Software Inc., Encinitas, CA, USA); rank correlation and U-tests by Statstica (StatSoft Inc, version 6.1, Tulsa, OK, USA); all other calculations were performed by Microsoft EXCEL (Microsoft Corp., Seattle, USA).

## 3 Results

For relating the biomagnetic susceptibility Δχ to LIC_dry wt_ (equation (2)), the wet-to-dry weight ratio f_wdr_ has to be known. A mean f_wdr_ of 4.10 ± 0.68 (range: 3.05–6.08) was obtained from the ratio of net weight of the fresh-tissue samples immediately after the biopsy procedure (2nd pass) and the dry weight after the heating procedure at MAYO. In 5 patients, biopsies were repeated after 17–36 months. In contrast to expected similar ratios between the f_wdr_ of the 1st and 2nd biopsy, it varied between 5 % and 59 % in the respective biopsy pairs.

The median LIC ± MAD from fresh-tissue biopsies (LIC_FT_) after drying was 10,833 ± 5674 μg/g_dry wt_ (range: 1854–32,864 μg/g) for patients with thalassemia or sickle cell disease (TDT&SCD) and for patients with hemochromatosis (HFE-1) after wet ashing it was 2107 ± 1521 μg/g_wet wt_ (range: 30–9760 μg/g).

The correlation between Δχ_liver_ measured by SQUID biosusceptometry and LIC_FT_ was non-parametrically tested in 38/40 patients (measurements) with TDT&SCD resulting in Spearman rank correlation coefficients of r_S_ = 0.91 (LIC_dry wt_) or r_S_ = 0.86 (LIC_wet wt_) both with p < 0.0001. In patients with HFE-1, a tight correlation with r_S_ = 0.98 (LIC_wet wt_) was found on the same significance level.

### 3.1 In vivo iron-specific magnetic liver susceptibilities (χ_Fe_) by wet weight biopsies

**Fig. 1:**
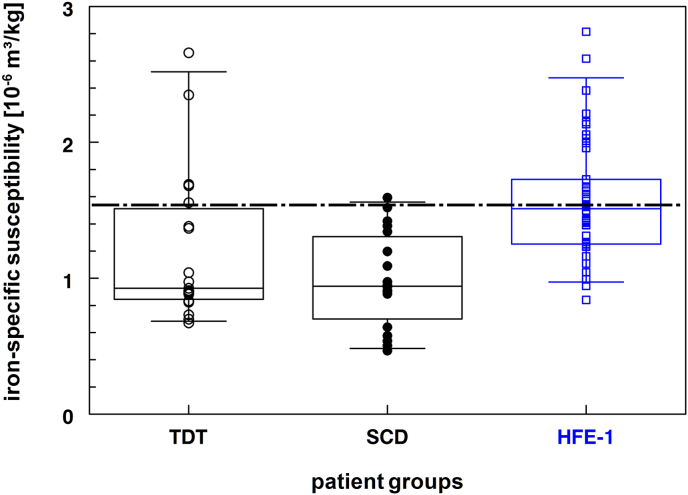
Box-whisker plot of iron-specific magnetic liver susceptibilities for patients with TDT, SCD, and HFE-1 with median values (within their interquartile range) of 0.93 (0.86–1.47), 0.94 (0.76–1.27), and 1.51 (1.25–1.69) **⋅** 10^−6^m^3^/kg, respectively. The dashed-dotted line indicates the traditional magnetic susceptibility for iron overload patients.

LIC_wet wt_, which is directly related to the bulk susceptibility Δχ (equation (2)), could be calculated from LIC_dry wt_ and individual wet-to-dry weight ratios (f_wdr_; range 3.2–5.6) for TDT/SCD patients. Thus, individual magnetic mass susceptibilities χ_Fe_ were calculated from equation (2). In Fig. 1 (see also Table 1), these individual magnetic susceptibilities are shown within box-plots for patients with thalassemia (TDT) and sickle cell disease (SCD) with median ± MAD values of (0.93 ± 0.21)**⋅**10^−6^ m^3^/kg and (0.94 ± 0.30)**⋅**10^−6^ m^3^/kg, respectively. In patients with hemochromatosis (HFE-1), individual χ_Fe_ values were calculated from equation (2) based on LIC_wet wt_ (consistent with f_wdr_ ≡ 1) as χ_Fe_ ± MAD of (1.51 ± 0.26)**⋅**10^−6^ m^3^/kg. The only significant difference was found for patients with HFE-1 (p < 0.003) when compared with TDT or SCD patients.

**Table 1:** Median values ± median absolute deviation (MAD) of demographic and of iron related data (LIC, biopsy weight, iron-specific magnetic mass susceptibility χ_Fe_) in patients with transfusion dependent thalassemia (TDT), sickle cell disease (SCD), and hereditary hemochromatosis (HFE-1).

Data	TDT	SCD	HFE-1^a^
n (female/male)	20 (8/12)	20 (11/9)	42 (11/27)
age (years)	20.5 ± 6.5	15.0 ± 2.8	45.9 ± 5.2
biopsy weight m_wet wt_ (mg)	10.9 ± 1.4	10.7 ± 1.2	14.9 ± 5.6
mean wet-to-dry weight ratio (f_wdr_) ± SD	4.08 ± 0.77	4.05 ± 0.63	1.0 b
traditional BLS-LIC {χ_Fe_ = 1.52 ppm} (μg/g_liver_)	1722 ± 783	1774 ± 416	2225 ± 1227
LIC (biopsy) (μg/g_wet wt_)	2805 ± 1595	2581 ± 762	2107 ± 1521
χ_Fe_ by wet-weight biopsies (10^−6^m^3^/kg)	0.93 ± 0.21	0.94 ± 0.30	1.51 ± 0.26^b^
novel χ_Fe_ (10^−6^m^3^/kg)^d^	0.96 ± 0.03^c^		1.48 ± 0.03^b^^,^^e^
SEE of volume susceptibility Δχ (ppb)	888		428^e^
LIC (BLS, novel χ_Fe_) (μg/g_liver_)	2726 ± 1175		2295 ± 1266

a 42 biopsies from 37 patients.

b from wet-weight biopsies only.

c from dry-weight biopsies.

d from weighted regression: coefficients ± standard error.

e LIC <5000 μg/g_wet wt_ (<20000 μg/g_dry wt_), SEE = standard error of estimate.

### 3.2 Averaged in vivo iron-specific magnetic liver susceptibility (χ_Fe_) by dry weight biopsies

Iron-specific magnetic liver susceptibilities (χ_Fe_) can also be calculated from the regression analysis of volume susceptibility differences by biosusceptometry (Δχ) versus dry weight LIC from fresh-tissue biopsies. According to equation (2), an averaged χ_Fe_ was derived from the slope, χ_Fe_ **·** 1.05/f_wdr_, of the regression lines.

**Fig. 2:**
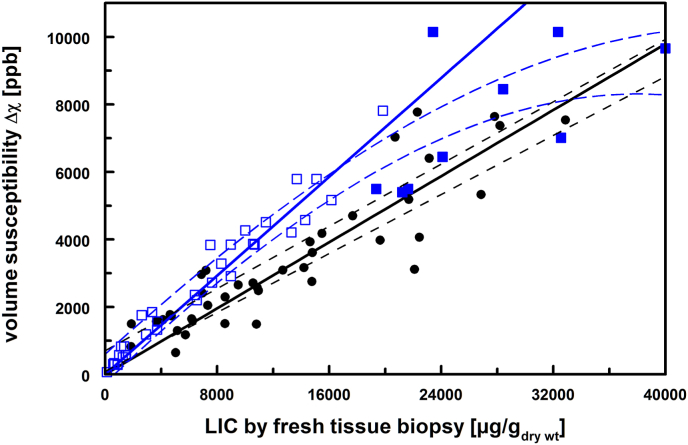
Magnetic volume susceptibility (Δχ) in patients with hemochromatosis (HFE-1, blue squares, f_wdr_ = 4.1) and thalassemia or sickle cell disease (TDT&SCD, black circles). Solid blue squares indicate HFE-1 patients with transformed LIC >20000 μg/g_dry wt_. From Δχ by biosusceptometry versus LIC from biopsies, the iron-specific susceptibility (χ_Fe_) can be calculated from the slopes of the weighted regression lines (blue: r^2^ = 0.99 and black: r^2^ = 0.89) as 1.43**·**10^−6^m^3^/kg and 0.96**·**10^−6^m^3^/kg for HFE-1 and TDT&SCD, respectively. Dashed lines indicate ±95 % confidence intervals from logistic and linear function fits to data in the whole LIC range.

In Fig. 2, LIC_wet wt_ of HFE-1 patients were transformed into dry weight LIC by the wet-to-dry weight ratio of 4.1 for better visual comparability with TDT&SCD patients. A nonlinear fit (Hill type) was applied to the 42 biopsy data resulting in a 95 % confidence interval (r^2^ = 0.92). Biopsies with LIC >20000 μg/g_dry wt_ were characterized by χ_Fe_ in the range of (0.8–1.7) **·**10^−6^ m^3^/kg and by higher hemosiderin iron fractions (>60 %) in 7/9 patients. Nearly all of these patients were affected by fibrosis, inflammation, or cirrhosis. Therefore, LIC was restricted to values < 20000 μg/g_dry wt_ (<5000 μg/g_wet wt_) in HFE-1 patients [9]. Since Δχ is also susceptible to uncertainties from ΔV_thorax_ especially in patients with BMI >30 kg/m^2^ or with low LIC, we applied weights calculated from uncertainties of Δχ to the linear regression fit.

The respective weighted regression analysis with a priori zero intercept resulted in χ_Fe_ = (1.48 ± 0.03)·10^−6^m^3^/kg (r^2^ = 0.99) and χ_Fe_ = (0.96 ± 0.03)·10^−6^m^3^/kg (r^2^ = 0.89) for patients with HFE-1 (LIC <20000 μg/g_dry wt_) and TDT&SCD, respectively. A potential intercept was small and erroneous for HFE-1 (201 ± 113 ppb, p = 0.08) and TDT&SCD (398 ± 260 ppb, p = 0.13) patients.

Using wet weight biopsies for TDT&SCD patients and weighted linear regression, we obtained χ_Fe_ = (0.87 ± 0.04) ·10^−6^ m^3^/kg, but with a smaller coefficient of determination (r^2^) of 0.83. Weighted linear regression analysis of Δχ and LIC from paraffin-embedded biopsies resulted in very similar values of χ_Fe_ = (0.89 ± 0.03) ·10^−6^m^3^/kg and slightly better r^2^ = 0.89 in patients with TDT&SCD.

**Fig. 3:**
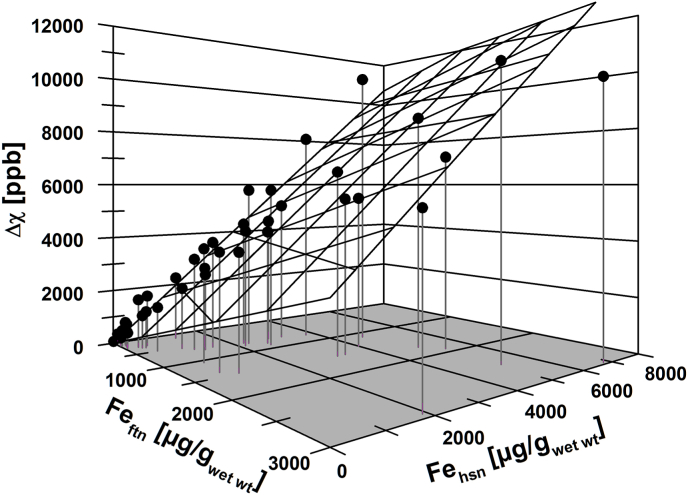
Bivariate regression analysis (gridded plane, r^2^ = 0.95) of magnetic volume susceptibility difference Δχ as function of ferritin-like (Fe_ftn_) and hemosiderin-like iron (Fe_hsn_) concentration in liver tissue of hemochromatosis patients (HFE-1) yielded partial mass susceptibilities of 1.01 ·10^−6^m^3^/kg and 1.27 ·10^−6^m^3^/kg for Fe_ftn_ and Fe_hsn_, respectively.

In HFE-1 patients, we investigated the nonparametric correlation between the three variables Δχ and the ferritin and hemosiderin iron concentrations (Fe_ftn_, Fe_hsn_) in more detail. The Spearman rank correlation coefficients resulted in r_S_ = 0.92 (p < 10^−6^) for Δχ vs. Fe_ftn_ and in r_S_ = 0.97 (p < 10^−6^) for Δχ vs. Fe_hsn_, respectively. Hemoglobin iron did not correlate with Δχ (p = 0.7). Using the Weidemann relation [14] in equation (2), χ_Fe_ can be split into a sum of ferritin and hemosiderin iron-specific susceptibilities. A bivariate regression analysis of Δχ = linear function (Fe_ftn_, Fe_hsn_) with zero intercept (r^2^ = 0.95, p < 10^−6^) resulted in significant partial mass susceptibilities of χ_ftn_ = (1.01 ± 0.30)·10^−6^m^3^/kg and χ_hsn_ = (1.27 ± 0.13)·10^−6^m^3^/kg (see Fig. 3).

The most important results are summarized in Table 1. Using the newly calculated iron-specific mass susceptibilities χ_Fe_ (novel), LIC by BLS will significantly increase for patients with transfusion dependent thalassemia and sickle cell disease, while for patients with hereditary hemochromatosis LIC does not significantly change.

## 4 Discussion

The magnetic volume susceptibility difference relative to a water reference (Δχ) is associated with liver iron concentration (LIC) by the wet-to-dry weight ratio (f_wdr_), the specific liver density (ρ), and the iron-specific paramagnetic mass susceptibility (χ_Fe_), see equation (2). There might be a potentially small effect from the iron-free liver tissue (Δχ_tissue_) [23], which mainly depends on the blood and fat concentration in liver tissue. However, there was no significant contribution in the order of 100–400 ppb which is in agreement with previous estimations [31].

The specific liver density was studied by Overmoyer et al. [30] in 21 autopsy livers by wedge (500–1000 mg) with nonheme iron content between 30 and 480 μg/g_liver_. Liver density, measured by saline displacement of wedge biopsies, of 1.051 ± 0.013 g/cm^3^ (range 1.017–1.077) did not correlate with the iron content. In a CT study of liver transplant patients (n = 99), a density of 1.03 g/cm^3^ could be calculated from the mean weight and volume of the excised livers [32].

Noninvasive iron measurement techniques (MRI, BLS) are volumetric methods with iron concentrations in wet weight biopsies (equation (2)). The outcomes from MRI (R2, R2∗, SIR) are usually compared with dry weight iron concentrations by biopsy [[11], [12], [13]]. We measured wet and dry weight in fresh-tissue biopsy samples weighing between 6 and 17 mg with a mean wet-to-dry weight ratio of 4.1 ± 0.7. This confirms a previous study of reference, Zuyderhoudt et al. [19], where a mean percentage of 24.6 % ± 3.7 % dry weight was evident in post-mortem wet weight biopsies, equivalent to a WDR of 4.07 ± 0.61.

The size of the biopsy could play a role in the WDR determination. In the study by Barry [18], the dry weight made up only 17 % of large wet weight biopsies (>30 mg) leading to a WDR of 5.9 which was comparable with a WDR of 5.5 ± 0.9 identified in large perinatal autopsy liver tissue samples (mean weight 68 ± 50 g) [33]. In non-human primates (marmosets), a WDR of 5.13 ± 0.6 was obtained from 22 autopsy tissue samples (100–500 mg) [34]. A higher WDR might be expected for larger samples due to relatively less humidity loss as compared with small biopsies.

In vivo total liver iron assessed by a room-temperature based magnetic iron detector (MID) was correlated with LIC from 26 dry weight biopsies (TDT (n = 13), HFE-1 (n = 6), and other hematological disorders (n = 7)). A wet-to-dry conversion factor C_wd_ = 5.8 (equation (2a)) between LIC from dry-weight biopsies and LIC from MID based on an iron-specific mass susceptibility of 1.45 ⋅10^−6^ m^3^/kg was evaluated [35]. Relating LIC_dry wt_ by MRI-R2∗ in 97 patients to LIC from MID revealed a conversion factor of 6.7 ± 0.4 [36]. The magnetic liver susceptibility derived from the MR phase (field) difference between two adjacent small lateral ROIs was compared with dry weight LIC from MRI-R2∗ measurements in the same liver vicinity (r^2^ = 0.94) [37]. Using a calculated (Curie's law) specific iron susceptibility of χ_Fe_ = 1.45∙10^−6^ m^3^/kg for liver iron [17], a C_wd_ of 8.99 ± 0.15 mgFe/g_dry wt_ per mgFe/mL of wet liver was received. This factor is even higher than the conversion factor of 6 (μg/g_dry wt_)/(μg/g_liver_) based on the “universal” iron-specific susceptibility of 1.524 ∙10^−6^ m^3^/kg [16,22].

Sharma et al. [28] compared BLS with MRI (3.0 T) based quantitative susceptibility mapping (MRI-QSM) in 22 patients (TDT: 50 %). The susceptibility difference Δχ from QSM between adjacent ROIs in subcutaneous body fat and right liver lobe resulted in half of the value by BLS (0.49 ± 0.05). More recently, Zhao et al. [38] used the SQUID facility at Oakland in order to compare Δχ from BLS and QSM in 19 iron overload patients (HFE-1: 42 %, TDTSCD: 26 %) at 1.5 and 3.0 T. They found a conversion factor of (0.60 ± 0.06) ppm_QSM_/ppm_BLS_ at 3.0 T. For both QSM techniques, there was an upper threshold for iron assessment at 4–6 ppm, i. e. 16-24 mg/g_dry wt_. In a murine model, iron was measured by QSM at 7.0 T in excised livers 8 days after parenteral administration of iron dextran. Comparison with physico-chemically (ICP-MS) determined iron resulted in a conversion factor of 0.83 ppm/(mg/g_wet wt_), which is close to the expected value of Δχ/LIC ≈ 1 [39].

Different processing techniques of liver biopsies by wet ashing in HFE-1 patients and by heat drying in TDT/SCD patients could have caused potential differences in the determination of iron-specific magnetic susceptibilities. Since MRI-R2∗ is closely related to χ_Fe_ (17), we may also use iron assessments by R2∗ in comparison to liver biopsy. In the framework of deferasirox studies, LIC data obtained from paraffin embedded biopsy samples were compared with SQUID-BLS and MRI-R2 from several centers [40]. LIC from BLS was related by a factor of 0.46 based on wet-to-dry weight ratio of f_wdr_ = 3.33. Using the more realistic f_wdr_ of 4.1 and the novel χ_Fe_ for TDT patients, one would have achieved a factor of 0.85. This agrees with the relation factor of 0.72 for the comparison with LIC from MRI-R2 within its confidence interval of 0.54–0.97.

Similar results were given in a study assessing the response to deferasirox treatment in transfused non-thalassemic patients by using LIC from BLS and biopsy (as above) [41]. A relation factor of 0.5 was observed.

In principle, differences between iron-specific magnetic susceptibilities should also be reflected in LIC assessed by R2∗ measurements of HFE-1 and TDT patients and characterized by LIC(R2∗) ∼ R2∗/χ_Fe_. Henninger et al. [42] compared LIC(R2∗) in 17 non-transfused patients (5/17 homozygous and compound heterozygous for HFE-1) with data from Garbowski et al. [43] and other centers. The differences in the regression slopes of 0.32 and 0.24 could be explained in part by the ratio of 1.5 between χ_Fe_ for HFE-1 and TDT patients.

Error propagation in equation (2) with typical patient values ± uncertainties for Δχ, LIC, f_wdr_, and ρ yielded a coefficient of variation (COV) of 21 % for dχ_Fe_/χ_Fe_ with the highest contribution from f_wdr_ (17 %). This may underline the importance of a precise wet-to-dry weight ratio.

Another limitation is the uneven iron distribution within the liver on the biopsy level as well as on the BLS or MRI-R2 level. In two severely iron loaded thalassemia patients, COVs of 14 % and 24 % were found [44]. MRI-R2 variations between 32 liver chunks (size 1 cm^3^) from an autopsy thalassemic liver slice was observed in the same range (COV = 19 %), while the intact liver slice revealed a variability of 29 % in R2 [45]. The patient with the highest Δχ in Fig. 2 had also a maximum COV of 23 % in two adjacent wet biopsies (5.7 and 7.9 mg_wet wt_).

The hemosiderin-ferritin iron metabolism was investigated in the past in biopsies from patients with HFE-1 (16/34) and iron-loading anemia patients (n = 14/34), not treated by phlebotomy or chelation [46]. A hemosiderin:ferritin iron ratio of about 1:1 at 1000 μg/g_liver_ and beyond was observed in contrast to the findings of Morgan and Walters [47] and in this work, where more iron was stored as hemosiderin than as ferritin above 1000 μg/g_liver_ (ratio 3:1) [17]. More recently, MRI-R2 of Carr-Purcell-Meiboom-Gill type with different interecho spacings was applied to differentiate between ferritin-like and hemosiderin-like (aggregated) liver iron in TDT patients with iron overload [48]. The aggregated iron linearly increased relative to total iron up to 90 % at a LIC >8000 μg/g_liver_.

From early on, ferritin-like and hemosiderin-like biominerals were studied by Mössbauer spectroscopy [49], electron transmission microscopy (TEM), electron diffraction, extended x-ray absorption, and AC magnetic susceptometry in iron overload animals and humans. A more recent study with low-temperature (5–60 K) Mössbauer spectroscopy in organs from ^57^Fe-enriched hemochromatosis mice could show the existence of an iron- and age-dependent threshold for generating hemosiderin earlier in spleens than in livers [50].

Splenic hemosiderin iron in the form of Goethite (α-FeOOH), detected as sextet fraction in Mössbauer spectra, was found to be higher in TDT patients than in NTDT patients [23,51]. A chelator induced mobilisation of iron from ferrihydrite core to form the Goethite-like form of hemosiderin might be the explanation. In their review, Ward et al. [52] presented data on hemosiderin iron cores, predominantly occurring as amorphous ferric oxide in hemochromatosis and as Goethite in thalassemia patients. Iron release from human spleen hemosiderin by deferroxamine was substantially less than that released from ferritin samples [53].

Rats receiving a single dose of iron dextran, were studied by TEM and AC susceptometry in a temperature range of 1.8 K – 300 K at different time points. At 30–84 days post sacrifice, decreasing liver iron concentrations together with increasing magnetic susceptibilities from 0.55**·**10^−6^m^3^/kg to 1.45**·**10^−6^m^3^/kg were observed [54].

From our results together with the work of former contributors to this field, we conclude that χ_Fe_ might depend more on the underlying iron overload disorder, blood transfusion regimen, chelation or phlebotomy treatment, and specifically, from the distribution of ferritin and hemosiderin iron rather than from the type of organ [23]. In mostly untreated HFE-1 patients (Fig. 3), the hemosiderin iron in the liver gives rise to a significantly higher magnetic susceptibility than the ferritin iron. Beyond the agreement of the ferritin iron-specific magnetic susceptibility with former findings [23], however, this relation may change with the passage of phlebotomy treatment.

## 5 Summary and conclusion

In this analysis, median values of liver iron-specific χ_Fe_ of 0.93**·**10^−6^m^3^/kg, 0.94**·**10^−6^m^3^/kg, and 1.53**·**10^−6^m^3^/kg were obtained from TDT, SCD, and HFE-1 patients, respectively. The median value and range was very similar in liver and spleen in the transfusion dependent (TDT, SCD) and non-transfusion dependent (HFE-1, NTDT) disease groups, although the techniques of AC small sample susceptometry and large sample BLS were different as mentioned before.

From the rank correlation and regression analysis of Δχ versus dry weight LIC, Spearman rank correlation coefficients (r_S_) and coefficients of determination (r^2^) were found to be superior to wet weight LIC. Thus, we would conclude that dry weight LIC is favorable for magnetic mass susceptibility determination if the wet-to-dry weight ratio is well known as in this study, where Zuyderhoudt's wet-to-dry weight ratio of 4.1 was confirmed.

Currently, most iron assessments in organs, specifically in the liver, are performed by MRI-R2∗ and MRI-R2. These assessments are based on linear and nonlinear calibration curves versus liver biopsies. Across current centers (13, 42, 43), a R2∗ = 600 s^−1^ would reveal a LIC of 16.6 ± 2.1 mg/g_dry wt_. In a clinical setting, the resulting LIC will not only depend on MR scan sequences, patient behavior, analysis techniques, curve parameters, and iron distribution within a selected liver region, but also from the iron-specific magnetic susceptibility of the patient's liver under investigation.

Dry weight conversion factors of about 6 (mg/g_dry wt_)/ppm in transfusion dependent patients instead of 4 (=f_wdr_) can be explained by compensating the “universal χ_Fe_“ by a factor of 1.5, see equations (2) and (2a). For hemochromatosis patients (HFE-1), the conversion of the magnetic liver volume susceptibility difference (Δχ) into wet weight LIC by a divisor of about 1.5 (mg/g_wet wt_)/ppm was endorsed. This may also be appropriate for patients with NTDT and probably for other iron loading anemias. For patients with TDT, SCD, and maybe other transfusion dependent hemoglobinopathies, the conversion factor is about 1.0 (mg/g_wet wt_)/ppm. This factor may also apply to the spleen and other organs loaded by transfused iron as well.

The resulting data show the relevance of using different iron-specific susceptibilities if noninvasive iron assessment techniques such as BLS or quantitative MRI are applied to different iron overload diseases.

## Author information

All authors have approved the submitted version and have agreed both to be personally accountable for the author's own contributions and to ensure that questions related to the accuracy or integrity of any part of the work, even ones in which the author was not personally involved, are appropriately investigated, resolved, and the resolution documented in the literature.

## Ethics declarations

This work has been carried out in accordance with Declaration of Helsinki for experiments involving humans.

## Financial interest

This project was supported in part by a small grant from Novartis Pharmaceuticals.

## Declaration of competing interest

The authors declare that they have no known competing financial interests or personal relationships that could have appeared to influence the work reported in this paper.

## Data Availability

Data will be made available on request.
